# Bis{3-amino-1-carbamo­thioyl-5-[(2-{[(5-methyl-1*H*-imidazol-3-ium-4-yl)meth­yl]sulfan­yl}eth­yl)amino]-1*H*-1,2,4-triazol-4-ium} hexa­chloridobismuthate(III) nitrate dihydrate

**DOI:** 10.1107/S1600536813016449

**Published:** 2013-06-19

**Authors:** G. M. Golzar Hossain

**Affiliations:** a Department of Chemistry, University of Dhaka, Dhaka 1000, Bangladesh

## Abstract

The asymmetric unit of the title hydrated salt, (C_10_H_18_N_8_S_2_)_2_[BiCl_6_]NO_3_·2H_2_O, contains two independent 3-amino-1-carbamo­thioyl-5-[(2-{[(5-methyl-1*H*-imidazol-3-ium-4-yl)meth­yl]sulfan­yl}eth­yl)amino]-1*H*-1,2,4-triazol-4-ium cations, one hexa­chloridobismuthate anion, one nitrate anion and two solvent water mol­ecules. The dihedral angles between the imidazole and triazole rings in the cations are 44.7 (3) and 89.4 (3)°. The Bi^III^ ion is coordinated by six chloride ligands in a slightly distorted octa­hedral geometry. In each cation, an intra­molecular N—H⋯S hydrogen bond is observed. In the crystal, N—H⋯Cl, N—H⋯S, N—H⋯O, O—H⋯Cl, O—H⋯S and O—H⋯O hydrogen bonds connect the components into a three-dimensional network. In addtion, π–π stacking inter­actions between inversion-related triazole rings are observed, with a centroid–centroid distance of 3.322 (3) Å

## Related literature

For background to hexa­chloridobismuthate(III) complexes with organic cations, see: Laza­rini (1987 ▶); Jarraya *et al.* (1993 ▶); Battaglia & Corradi (1986 ▶); Bednarska-Bolek *et al.* (2000 ▶).
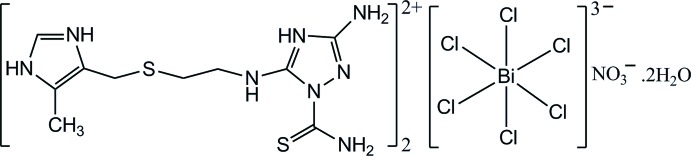


## Experimental

### 

#### Crystal data

(C_10_H_18_N_8_S_2_)_2_[BiCl_6_]NO_3_·2H_2_O
*M* *_r_* = 1148.61
Triclinic, 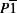

*a* = 8.8750 (1) Å
*b* = 14.2860 (2) Å
*c* = 16.6500 (2) Å
α = 94.376 (1)°
β = 100.717 (1)°
γ = 92.167 (1)°
*V* = 2065.24 (4) Å^3^
*Z* = 2
Mo *K*α radiation
μ = 4.91 mm^−1^
*T* = 150 K
0.20 × 0.20 × 0.20 mm

#### Data collection

Nonius KappaCCD diffractometer
Absorption correction: multi-scan (*SORTAV*; Blessing, 1995 ▶) *T*_min_ = 0.440, *T*_max_ = 0.440
34173 measured reflections
9432 independent reflections
8522 reflections with *I* > 2σ(*I*)
*R*_int_ = 0.061

#### Refinement

*R*[*F* ^2^ > 2σ(*F* ^2^)] = 0.040
*wR*(*F* ^2^) = 0.102
*S* = 1.05
9432 reflections
541 parameters
24 restraints
Δρ_max_ = 2.72 e Å^−3^
Δρ_min_ = −2.16 e Å^−3^

### 

Data collection: *DENZO* (Otwinowski & Minor, 1997 ▶) and *COLLECT* (Hooft, 1998 ▶); cell refinement: *DENZO* and *COLLECT*; data reduction: *DENZO* and *COLLECT*; program(s) used to solve structure: *SHELXS97* (Sheldrick, 2008 ▶); program(s) used to refine structure: *SHELXL97* (Sheldrick, 2008 ▶); molecular graphics: *ORTEP-3 for Windows* (Farrugia, 2012 ▶) and *PLATON* (Spek, 2009 ▶); software used to prepare material for publication: *WinGX* (Farrugia, 2012 ▶).

## Supplementary Material

Crystal structure: contains datablock(s) global, I. DOI: 10.1107/S1600536813016449/lh5614sup1.cif

Structure factors: contains datablock(s) I. DOI: 10.1107/S1600536813016449/lh5614Isup2.hkl

Additional supplementary materials:  crystallographic information; 3D view; checkCIF report

## Figures and Tables

**Table 1:** Hydrogen-bond geometry (Å, °)

*D*—H⋯*A*	*D*—H	H⋯*A*	*D*⋯*A*	*D*—H⋯*A*
N1—H1*N*⋯Cl6^i^	0.89 (4)	2.54 (3)	3.379 (6)	158 (7)
N2—H2*N*⋯Cl6^ii^	0.88 (5)	2.58 (5)	3.406 (5)	156 (6)
N3—H3*N*⋯S2	0.89 (5)	2.23 (6)	3.019 (6)	148 (7)
N4—H4*N*⋯O2	0.91 (7)	1.74 (7)	2.608 (7)	160 (7)
N7—H7*A*⋯S2^iii^	0.91 (6)	2.46 (7)	3.296 (6)	155 (6)
N7—H7*B*⋯O1^iv^	0.90 (4)	1.97 (5)	2.842 (8)	162 (6)
N8—H8*A*⋯Cl5^iv^	0.89 (5)	2.83 (6)	3.550 (5)	140 (5)
O1—H11⋯Cl5	0.84 (6)	2.39 (6)	3.138 (5)	149 (6)
N11—H11*N*⋯O1^v^	0.89 (2)	1.83 (3)	2.703 (6)	170 (9)
O1—H12⋯Cl4^vi^	0.85 (6)	2.27 (5)	3.116 (5)	172 (7)
N12—H12*N*⋯O3^ii^	0.88 (6)	1.99 (7)	2.835 (6)	162 (6)
N12—H12*N*⋯O4^ii^	0.88 (6)	2.58 (6)	3.307 (8)	142 (5)
N13—H13*N*⋯S4	0.88 (3)	2.27 (6)	3.025 (4)	144 (7)
N14—H14*N*⋯O3^vii^	0.87 (6)	1.91 (6)	2.772 (5)	175 (5)
N14—H14*N*⋯O5^vii^	0.87 (6)	2.43 (8)	2.960 (7)	120 (7)
N17—H17*A*⋯S4^iii^	0.87 (6)	2.60 (6)	3.423 (5)	159 (6)
N17—H17*B*⋯Cl3^iii^	0.87 (3)	2.52 (5)	3.337 (5)	158 (6)
N18—H18*A*⋯Cl2	0.87 (5)	2.56 (7)	3.304 (5)	144 (5)
N18—H18*B*⋯Cl3	0.88 (4)	2.59 (7)	3.332 (5)	143 (7)
O2—H21⋯Cl4	0.86 (4)	2.62 (6)	3.326 (5)	139 (6)
O2—H21⋯S2^iii^	0.86 (4)	2.59 (8)	3.168 (5)	126 (7)
O2—H22⋯O4	0.86 (3)	2.52 (5)	3.227 (7)	139 (7)
O2—H22⋯O5	0.86 (3)	1.88 (5)	2.717 (6)	162 (9)

Symmetry codes: (i) 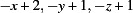; (ii) 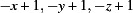; (iii) 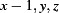; (iv) 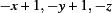; (v) 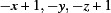; (vi) 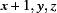; (vii) 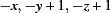.

**Fig. 1.**
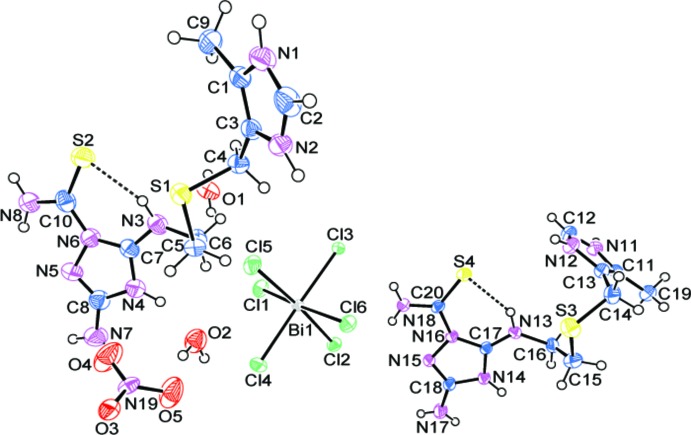
The molecular structure of the title compound showing 30% dispalcement ellipsoids (arbitrary spheres for the H atoms). Intramolecular hydrogen bonds are shown as dashed lines.

**Fig. 2.**
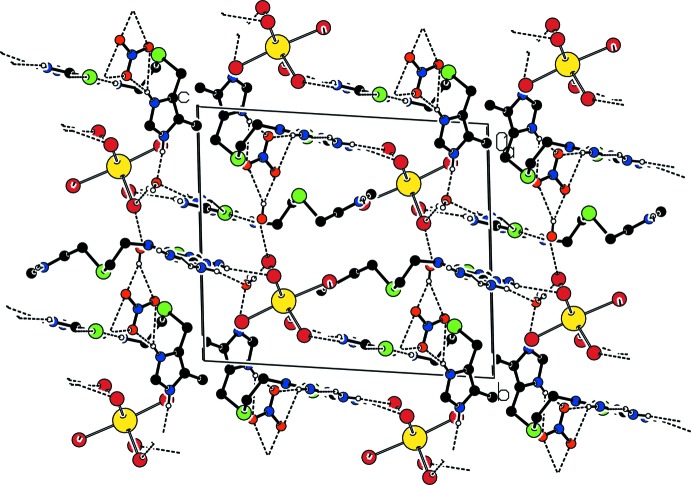
Packing diagram of the complex. Hydrogen bonds are shown by dashed lines.

## supplementary crystallographic information

### Comment

The hexachlorobismuthate(III) anion, [BiCl_6_]^3–^, forms complexes with organic cations in the presence of excess chloride ions (Lazarini, 1987; Jarraya *et al.*, 1993; Battaglia & Corradi, 1986). Cimetidine, *N*-cyano-*N*'-methyl-*N*"-{2-[5-methy-1*H*-imidazole-4-yl]methylthio-ethyl}-guanidine, consists of three main functional units: a 4,5-disubstituted imidazolic ring, a cysteine and an *N*-cyanoazamethine or guanidine derivative. The *N*-cyanoazamethine part of cimetidine reacts with thiosemicarbazide when the reaction is carried out with Bi(NO_3_)_3_ in presence of hydrochloric acid. The crystal structure of the title compound is presented herein.

The molecular structure of the title compound is shown in Fig. 1. The asymmetric unit compound, contains of two dicationic ligands, one [BiCl_6_]^3–^ anion, one NO_3_^-^ anion and two solvent water molecules. The Bi^III^ ion is coordinated by six chloride ions forming a slightly distorted octahedral configuration with Bi—Cl bond lengths ranging from 2.6312 (13) to 2.7984 (12)Å. The Bi–Cl bond lengths are comparable with the reported values in a related halogenobismuthate (Bednarska-Bolek *et al.*, 2000). The bond angles within the [BiCl_6_]^3–^ anion of the compound do not suggest any steric interactions are present (Bednarska-Bolek *et al.*, 2000; Jarraya *et al.*, 1993). The dihedral angles between the imidazole and triazole rings in the cations are 44.7 (3) [C1-C3/N1/N2 and N4-N6/C7/C8] and 89.4 (3)° [C11-C13/N11/N12 and C17/C18/N14-N16]. In each cation, an intramolecular N—H···S hydrogen bond is observed. In the crystal, N—H···Cl, N—H···S, N—H···O, O—H···Cl, O—H···S and O—H···O hydrogen bonds connect the components of the structure into a three-dimensional network (Fig. 2). In addtion, π–π stacking interactions with Cg···Cg(1-x, 1-y, -z) = 3.322 (3)Å are observed [Cg is the centroid of the N4/C7/N6/N5/C8 ring].

### Experimental

Bismuth nitrate (0.4851 g, 1.0 mmol) was dissolved in 4*M* hydrochloric acid (30 ml). An ethanolic solution of cimetidine (0.5047 g, 2.0 mmol) and thiosemicarbazide (0.1823 g, 2.0 mmol) were added slowly to the solution with constant stirring. The solution was stirred further for 3 h and then it was filtered and left with slow evaporation of the solvent. Colorless crystals suitable for X-ray analysis separated out from the solution after a week.

### Refinement

H atoms bonded to C atoms were placed in calculated positions [C—H = 0.95 Å, 0.98 Å and 0.99 Å] and refined in a riding-model approximation with U_iso_(H) = 1.2U_eq_(C) or U_iso_(H) = 1.2U_eq_(C_methyl_). H atoms bonded to N and O atoms were refined independently with a common isotropic displacement parameter for each type.

### Crystal data

**Table:**

(C_10_H_18_N_8_S_2_)_2_[BiCl_6_]NO_3_·2H_2_O	*Z* = 2
*M**_r_* = 1148.61	*F*(000) = 1136
Triclinic, *P*1	*D*_x_ = 1.850 Mg m^−^^3^
Hall symbol: -P 1	Mo *K*α radiation, λ = 0.71073 Å
*a* = 8.8750 (1) Å	Cell parameters from 9432 reflections
*b* = 14.2860 (2) Å	θ = 2.7–27.5°
*c* = 16.6500 (2) Å	µ = 4.91 mm^−^^1^
α = 94.376 (1)°	*T* = 150 K
β = 100.717 (1)°	Block, colorless
γ = 92.167 (1)°	0.20 × 0.20 × 0.20 mm
*V* = 2065.24 (4) Å^3^	

### Data collection

**Table:**

Nonius KappaCCD diffractometer	9432 independent reflections
Radiation source: fine-focus sealed tube	8522 reflections with *I* > 2σ(*I*)
Graphite monochromator	*R*_int_ = 0.061
ω scans	θ_max_ = 27.5°, θ_min_ = 3.0°
Absorption correction: multi-scan (*SORTAV*; Blessing, 1995)	*h* = −11→11
*T*_min_ = 0.440, *T*_max_ = 0.440	*k* = −18→18
34173 measured reflections	*l* = −21→21

### Refinement

**Table:**

Refinement on *F*^2^	Secondary atom site location: difference Fourier map
Least-squares matrix: full	Hydrogen site location: inferred from neighbouring sites
*R*[*F*^2^ > 2σ(*F*^2^)] = 0.040	H atoms treated by a mixture of independent and constrained refinement
*wR*(*F*^2^) = 0.102	*w* = 1/[σ^2^(*F*_o_^2^) + (0.0527*P*)^2^ + 5.0184*P*] where *P* = (*F*_o_^2^ + 2*F*_c_^2^)/3
*S* = 1.05	(Δ/σ)_max_ = 0.001
9432 reflections	Δρ_max_ = 2.72 e Å^−^^3^
541 parameters	Δρ_min_ = −2.16 e Å^−^^3^
24 restraints	Extinction correction: *SHELXL97* (Sheldrick, 2008), Fc^*^=kFc[1+0.001xFc^2^λ^3^/sin(2θ)]^-1/4^
Primary atom site location: structure-invariant direct methods	Extinction coefficient: 0.0014 (3)

### Special details

**Table:**

Geometry. All e.s.d.'s (except the e.s.d. in the dihedral angle between two l.s. planes) are estimated using the full covariance matrix. The cell e.s.d.'s are taken into account individually in the estimation of e.s.d.'s in distances, angles and torsion angles; correlations between e.s.d.'s in cell parameters are only used when they are defined by crystal symmetry. An approximate (isotropic) treatment of cell e.s.d.'s is used for estimating e.s.d.'s involving l.s. planes.
Refinement. Refinement of *F*^2^ against ALL reflections. The weighted *R*-factor *wR* and goodness of fit *S* are based on *F*^2^, conventional *R*-factors *R* are based on *F*, with *F* set to zero for negative *F*^2^. The threshold expression of *F*^2^ > σ(*F*^2^) is used only for calculating *R*-factors(gt) *etc*. and is not relevant to the choice of reflections for refinement. *R*-factors based on *F*^2^ are statistically about twice as large as those based on *F*, and *R*- factors based on ALL data will be even larger.

### Fractional atomic coordinates and isotropic or equivalent isotropic displacement parameters (Å^2^)

**Table:**

	*x*	*y*	*z*	*U*_iso_*/*U*_eq_	
S1	0.94732 (15)	0.67239 (9)	0.34141 (8)	0.0342 (3)	
S2	0.98090 (19)	0.58775 (13)	0.08391 (10)	0.0496 (4)	
N1	1.3722 (6)	0.6664 (4)	0.5461 (3)	0.0407 (11)	
N2	1.1338 (5)	0.6703 (3)	0.5467 (3)	0.0362 (10)	
N3	0.7375 (6)	0.5559 (4)	0.1852 (3)	0.0397 (11)	
N4	0.4915 (6)	0.5895 (4)	0.1124 (3)	0.0395 (11)	
N5	0.5433 (6)	0.6162 (4)	−0.0108 (3)	0.0416 (11)	
N6	0.6766 (5)	0.5934 (3)	0.0442 (3)	0.0381 (10)	
N7	0.2906 (7)	0.6302 (5)	0.0070 (4)	0.0582 (15)	
N8	0.8045 (6)	0.6170 (4)	−0.0581 (3)	0.0488 (13)	
C1	1.2997 (6)	0.6179 (4)	0.4738 (3)	0.0327 (11)	
C2	1.2710 (8)	0.6972 (5)	0.5884 (4)	0.0447 (14)	
H2	1.2929	0.7327	0.6402	0.054*	
C3	1.1469 (6)	0.6205 (4)	0.4735 (3)	0.0307 (10)	
C4	1.0117 (6)	0.5830 (4)	0.4118 (3)	0.0321 (11)	
H4A	0.9275	0.5644	0.4398	0.038*	
H4B	1.0389	0.5265	0.3804	0.038*	
C5	0.7434 (6)	0.6431 (4)	0.3189 (4)	0.0369 (12)	
H5A	0.7072	0.6377	0.3712	0.044*	
H5B	0.6905	0.6953	0.2917	0.044*	
C6	0.6977 (6)	0.5536 (4)	0.2652 (3)	0.0397 (13)	
H6A	0.7485	0.5010	0.2927	0.048*	
H6B	0.5853	0.5412	0.2585	0.048*	
C7	0.6429 (6)	0.5785 (4)	0.1187 (3)	0.0354 (12)	
C8	0.4379 (7)	0.6133 (4)	0.0328 (4)	0.0430 (13)	
C9	1.3858 (7)	0.5787 (5)	0.4114 (4)	0.0427 (13)	
H9A	1.4099	0.6285	0.3777	0.064*	
H9B	1.3230	0.5280	0.3764	0.064*	
H9C	1.4814	0.5539	0.4391	0.064*	
C10	0.8161 (7)	0.6009 (4)	0.0190 (4)	0.0424 (13)	
S3	0.24517 (18)	0.19615 (10)	0.86591 (9)	0.0416 (3)	
S4	0.32138 (14)	0.09271 (11)	0.62576 (8)	0.0352 (3)	
N11	0.3547 (5)	−0.1218 (3)	0.8769 (3)	0.0345 (10)	
N12	0.4499 (5)	0.0078 (4)	0.8468 (3)	0.0376 (10)	
N13	0.0633 (4)	0.0708 (3)	0.7198 (2)	0.0281 (9)	
N14	−0.1794 (5)	0.0852 (3)	0.6379 (2)	0.0270 (9)	
N15	−0.1160 (4)	0.1080 (3)	0.5168 (2)	0.0277 (9)	
N16	0.0146 (4)	0.0950 (3)	0.5773 (2)	0.0239 (8)	
N17	−0.3771 (5)	0.1081 (5)	0.5248 (3)	0.0467 (13)	
N18	0.1552 (5)	0.1189 (3)	0.4797 (3)	0.0315 (9)	
C11	0.3124 (6)	−0.0522 (4)	0.9296 (3)	0.0297 (10)	
C12	0.4370 (6)	−0.0842 (4)	0.8284 (3)	0.0383 (12)	
H12A	0.4798	−0.1175	0.7871	0.046*	
C13	0.3730 (6)	0.0303 (4)	0.9106 (3)	0.0313 (11)	
C14	0.3565 (7)	0.1286 (4)	0.9417 (3)	0.0368 (12)	
H14A	0.4600	0.1600	0.9599	0.044*	
H14B	0.3064	0.1282	0.9900	0.044*	
C15	0.0509 (6)	0.1450 (5)	0.8563 (4)	0.0421 (13)	
H15A	0.0348	0.1296	0.9112	0.051*	
H15B	−0.0230	0.1925	0.8373	0.051*	
C16	0.0160 (6)	0.0566 (4)	0.7974 (3)	0.0314 (11)	
H16A	0.0706	0.0038	0.8226	0.038*	
H16B	−0.0956	0.0398	0.7872	0.038*	
C17	−0.0274 (5)	0.0827 (3)	0.6512 (3)	0.0222 (9)	
C18	−0.2286 (6)	0.1011 (4)	0.5559 (3)	0.0304 (11)	
C19	0.2162 (6)	−0.0740 (5)	0.9913 (3)	0.0402 (13)	
H19A	0.2623	−0.1235	1.0239	0.060*	
H19B	0.1125	−0.0955	0.9628	0.060*	
H19C	0.2108	−0.0173	1.0274	0.060*	
C20	0.1598 (5)	0.1032 (3)	0.5573 (3)	0.0253 (9)	
Bi1	0.200467 (18)	0.255209 (11)	0.278175 (10)	0.02225 (8)	
Cl1	0.16339 (17)	0.16319 (11)	0.13224 (8)	0.0416 (3)	
Cl2	−0.05587 (14)	0.17031 (9)	0.30498 (8)	0.0328 (3)	
Cl3	0.38963 (14)	0.12542 (9)	0.34520 (7)	0.0301 (3)	
Cl4	0.03748 (16)	0.40620 (10)	0.23050 (9)	0.0392 (3)	
Cl5	0.44926 (16)	0.35260 (11)	0.24484 (9)	0.0454 (3)	
Cl6	0.24345 (15)	0.34255 (9)	0.43761 (8)	0.0341 (3)	
O3	0.3595 (4)	0.8967 (3)	0.2430 (2)	0.0373 (9)	
O4	0.4022 (7)	0.7788 (4)	0.1648 (4)	0.0764 (17)	
O5	0.2846 (7)	0.7574 (3)	0.2640 (4)	0.0723 (16)	
N19	0.3509 (5)	0.8126 (3)	0.2230 (3)	0.0330 (9)	
O1	0.7188 (6)	0.3082 (3)	0.1521 (3)	0.0549 (12)	
O2	0.2936 (6)	0.5740 (3)	0.2091 (3)	0.0565 (12)	
H1N	1.473 (3)	0.679 (5)	0.560 (5)	0.068*	
H2N	1.049 (5)	0.673 (5)	0.567 (4)	0.068*	
H3N	0.831 (4)	0.569 (6)	0.176 (5)	0.068*	
H4N	0.436 (8)	0.573 (6)	0.150 (4)	0.068*	
H7A	0.226 (7)	0.627 (6)	0.043 (4)	0.068*	
H7B	0.270 (9)	0.641 (6)	−0.0466 (18)	0.068*	
H8A	0.711 (4)	0.629 (6)	−0.084 (4)	0.068*	
H8B	0.898 (4)	0.623 (6)	−0.070 (5)	0.068*	
H11N	0.341 (9)	−0.1839 (15)	0.871 (5)	0.068*	
H12N	0.498 (8)	0.048 (4)	0.822 (4)	0.068*	
H13N	0.160 (3)	0.073 (6)	0.715 (5)	0.068*	
H14N	−0.232 (7)	0.089 (6)	0.677 (3)	0.068*	
H17A	−0.446 (7)	0.088 (5)	0.551 (4)	0.068*	
H17B	−0.414 (8)	0.110 (6)	0.4731 (16)	0.068*	
H18A	0.068 (5)	0.122 (6)	0.446 (4)	0.068*	
H18B	0.246 (4)	0.127 (6)	0.467 (5)	0.068*	
H11	0.646 (5)	0.338 (5)	0.165 (5)	0.085*	
H12	0.803 (4)	0.334 (5)	0.179 (5)	0.085*	
H21	0.199 (4)	0.554 (5)	0.203 (6)	0.085*	
H22	0.292 (8)	0.6343 (15)	0.216 (6)	0.085*	

### Atomic displacement parameters (Å^2^)

**Table:**

	*U*^11^	*U*^22^	*U*^33^	*U*^12^	*U*^13^	*U*^23^
S1	0.0337 (7)	0.0303 (7)	0.0368 (7)	−0.0021 (5)	0.0027 (5)	0.0041 (5)
S2	0.0429 (8)	0.0642 (11)	0.0432 (8)	0.0111 (7)	0.0087 (7)	0.0089 (8)
N1	0.034 (2)	0.047 (3)	0.038 (3)	0.005 (2)	0.001 (2)	0.002 (2)
N2	0.039 (3)	0.039 (3)	0.033 (2)	0.014 (2)	0.012 (2)	0.000 (2)
N3	0.038 (3)	0.039 (3)	0.039 (3)	0.000 (2)	−0.001 (2)	0.004 (2)
N4	0.039 (3)	0.039 (3)	0.039 (3)	0.004 (2)	0.006 (2)	0.002 (2)
N5	0.037 (3)	0.042 (3)	0.044 (3)	0.006 (2)	0.002 (2)	0.003 (2)
N6	0.036 (2)	0.044 (3)	0.033 (2)	0.002 (2)	0.0037 (19)	0.003 (2)
N7	0.041 (3)	0.082 (4)	0.051 (3)	0.014 (3)	0.003 (3)	0.013 (3)
N8	0.044 (3)	0.061 (4)	0.044 (3)	0.009 (3)	0.009 (2)	0.016 (3)
C1	0.032 (3)	0.034 (3)	0.032 (3)	0.005 (2)	0.005 (2)	0.005 (2)
C2	0.055 (4)	0.050 (4)	0.027 (3)	0.014 (3)	0.003 (3)	−0.004 (2)
C3	0.034 (3)	0.028 (3)	0.031 (3)	0.007 (2)	0.007 (2)	0.004 (2)
C4	0.031 (3)	0.026 (3)	0.039 (3)	0.002 (2)	0.006 (2)	0.001 (2)
C5	0.030 (3)	0.041 (3)	0.042 (3)	0.007 (2)	0.009 (2)	0.004 (2)
C6	0.033 (3)	0.047 (3)	0.037 (3)	−0.006 (2)	0.000 (2)	0.011 (3)
C7	0.039 (3)	0.026 (3)	0.040 (3)	0.001 (2)	0.004 (2)	0.001 (2)
C8	0.043 (3)	0.046 (3)	0.039 (3)	0.004 (3)	0.004 (3)	0.003 (3)
C9	0.043 (3)	0.052 (4)	0.038 (3)	0.009 (3)	0.017 (3)	0.004 (3)
C10	0.053 (4)	0.035 (3)	0.039 (3)	0.004 (3)	0.008 (3)	0.003 (2)
S3	0.0484 (8)	0.0323 (7)	0.0426 (8)	0.0018 (6)	0.0043 (6)	0.0042 (6)
S4	0.0211 (6)	0.0537 (8)	0.0322 (6)	0.0046 (5)	0.0071 (5)	0.0069 (6)
N11	0.036 (2)	0.035 (2)	0.034 (2)	0.0098 (19)	0.0080 (19)	0.002 (2)
N12	0.034 (2)	0.047 (3)	0.035 (2)	0.001 (2)	0.0139 (19)	0.008 (2)
N13	0.0219 (19)	0.038 (2)	0.025 (2)	0.0053 (17)	0.0041 (16)	0.0081 (18)
N14	0.022 (2)	0.036 (2)	0.024 (2)	0.0007 (16)	0.0057 (16)	0.0053 (17)
N15	0.0228 (19)	0.038 (2)	0.025 (2)	0.0043 (17)	0.0064 (16)	0.0087 (18)
N16	0.0197 (18)	0.028 (2)	0.0250 (19)	−0.0008 (15)	0.0055 (15)	0.0045 (16)
N17	0.025 (2)	0.084 (4)	0.035 (3)	0.009 (2)	0.009 (2)	0.017 (3)
N18	0.027 (2)	0.039 (3)	0.032 (2)	0.0053 (19)	0.0112 (18)	0.0116 (19)
C11	0.025 (2)	0.040 (3)	0.023 (2)	0.001 (2)	0.0019 (19)	0.005 (2)
C12	0.039 (3)	0.048 (3)	0.029 (3)	0.011 (2)	0.009 (2)	−0.003 (2)
C13	0.028 (2)	0.039 (3)	0.027 (2)	0.004 (2)	0.006 (2)	0.004 (2)
C14	0.044 (3)	0.037 (3)	0.028 (3)	0.000 (2)	0.004 (2)	−0.001 (2)
C15	0.034 (3)	0.052 (4)	0.042 (3)	0.009 (3)	0.011 (2)	0.002 (3)
C16	0.028 (2)	0.043 (3)	0.024 (2)	0.003 (2)	0.0054 (19)	0.010 (2)
C17	0.025 (2)	0.020 (2)	0.024 (2)	0.0003 (17)	0.0088 (18)	0.0030 (17)
C18	0.025 (2)	0.042 (3)	0.026 (2)	0.002 (2)	0.0066 (19)	0.006 (2)
C19	0.036 (3)	0.054 (4)	0.035 (3)	0.006 (3)	0.014 (2)	0.009 (3)
C20	0.025 (2)	0.024 (2)	0.030 (2)	−0.0001 (18)	0.0120 (19)	0.0038 (19)
Bi1	0.02270 (10)	0.02247 (11)	0.02244 (11)	0.00348 (6)	0.00593 (6)	0.00206 (6)
Cl1	0.0499 (8)	0.0454 (8)	0.0284 (6)	−0.0034 (6)	0.0096 (6)	−0.0064 (6)
Cl2	0.0273 (6)	0.0376 (7)	0.0335 (6)	−0.0020 (5)	0.0070 (5)	0.0013 (5)
Cl3	0.0298 (6)	0.0349 (6)	0.0288 (6)	0.0121 (5)	0.0096 (5)	0.0087 (5)
Cl4	0.0394 (7)	0.0322 (7)	0.0489 (8)	0.0130 (5)	0.0111 (6)	0.0099 (6)
Cl5	0.0355 (7)	0.0524 (9)	0.0486 (8)	−0.0115 (6)	0.0085 (6)	0.0105 (7)
Cl6	0.0362 (6)	0.0353 (7)	0.0301 (6)	−0.0006 (5)	0.0084 (5)	−0.0050 (5)
O3	0.034 (2)	0.036 (2)	0.045 (2)	0.0017 (16)	0.0146 (17)	0.0069 (17)
O4	0.104 (4)	0.052 (3)	0.089 (4)	−0.002 (3)	0.065 (4)	−0.011 (3)
O5	0.109 (4)	0.037 (3)	0.084 (4)	−0.009 (3)	0.056 (3)	0.005 (3)
N19	0.032 (2)	0.037 (3)	0.033 (2)	0.0059 (18)	0.0158 (18)	−0.0033 (19)
O1	0.049 (3)	0.048 (3)	0.063 (3)	0.009 (2)	−0.003 (2)	0.002 (2)
O2	0.065 (3)	0.048 (3)	0.060 (3)	−0.003 (2)	0.022 (3)	0.004 (2)

### Geometric parameters (Å, º)

**Table:**

S1—C5	1.805 (5)	N12—C13	1.388 (7)
S1—C4	1.834 (5)	N12—H12N	0.88 (2)
S2—C10	1.676 (6)	N13—C17	1.296 (6)
N1—C2	1.310 (8)	N13—C16	1.456 (6)
N1—C1	1.376 (7)	N13—H13N	0.87 (2)
N1—H1N	0.88 (2)	N14—C17	1.328 (6)
N2—C2	1.312 (8)	N14—C18	1.391 (6)
N2—C3	1.389 (7)	N14—H14N	0.87 (2)
N2—H2N	0.88 (2)	N15—C18	1.296 (6)
N3—C7	1.327 (7)	N15—N16	1.415 (5)
N3—C6	1.443 (7)	N16—C17	1.372 (6)
N3—H3N	0.89 (2)	N16—C20	1.393 (6)
N4—C7	1.345 (7)	N17—C18	1.333 (7)
N4—C8	1.393 (8)	N17—H17A	0.87 (2)
N4—H4N	0.90 (2)	N17—H17B	0.86 (2)
N5—C8	1.288 (8)	N18—C20	1.320 (6)
N5—N6	1.421 (7)	N18—H18A	0.87 (2)
N6—C7	1.359 (7)	N18—H18B	0.88 (2)
N6—C10	1.382 (8)	C11—C13	1.360 (7)
N7—C8	1.336 (8)	C11—C19	1.494 (7)
N7—H7A	0.90 (2)	C12—H12A	0.9500
N7—H7B	0.90 (2)	C13—C14	1.482 (8)
N8—C10	1.308 (8)	C14—H14A	0.9900
N8—H8A	0.89 (2)	C14—H14B	0.9900
N8—H8B	0.89 (2)	C15—C16	1.523 (8)
C1—C3	1.356 (7)	C15—H15A	0.9900
C1—C9	1.488 (8)	C15—H15B	0.9900
C2—H2	0.9500	C16—H16A	0.9900
C3—C4	1.480 (7)	C16—H16B	0.9900
C4—H4A	0.9900	C19—H19A	0.9800
C4—H4B	0.9900	C19—H19B	0.9800
C5—C6	1.499 (8)	C19—H19C	0.9800
C5—H5A	0.9900	Bi1—Cl1	2.6312 (13)
C5—H5B	0.9900	Bi1—Cl2	2.6641 (12)
C6—H6A	0.9900	Bi1—Cl3	2.7122 (11)
C6—H6B	0.9900	Bi1—Cl4	2.7286 (13)
C9—H9A	0.9800	Bi1—Cl5	2.7314 (13)
C9—H9B	0.9800	Bi1—Cl6	2.7984 (12)
C9—H9C	0.9800	O3—N19	1.218 (6)
S3—C14	1.815 (6)	O4—N19	1.220 (6)
S3—C15	1.822 (6)	O5—N19	1.276 (6)
S4—C20	1.677 (5)	O1—H11	0.85 (2)
N11—C12	1.315 (7)	O1—H12	0.85 (2)
N11—C11	1.385 (7)	O2—H21	0.86 (2)
N11—H11N	0.89 (2)	O2—H22	0.86 (2)
N12—C12	1.323 (8)		
			
C5—S1—C4	100.1 (3)	C17—N14—C18	107.7 (4)
C2—N1—C1	110.3 (5)	C17—N14—H14N	123 (5)
C2—N1—H1N	125 (5)	C18—N14—H14N	128 (5)
C1—N1—H1N	125 (5)	C18—N15—N16	103.3 (4)
C2—N2—C3	109.7 (5)	C17—N16—C20	130.2 (4)
C2—N2—H2N	124 (5)	C17—N16—N15	110.6 (4)
C3—N2—H2N	125 (5)	C20—N16—N15	119.0 (4)
C7—N3—C6	124.6 (5)	C18—N17—H17A	120 (5)
C7—N3—H3N	105 (5)	C18—N17—H17B	124 (5)
C6—N3—H3N	125 (5)	H17A—N17—H17B	111 (7)
C7—N4—C8	106.4 (5)	C20—N18—H18A	122 (5)
C7—N4—H4N	124 (5)	C20—N18—H18B	114 (5)
C8—N4—H4N	128 (5)	H18A—N18—H18B	124 (7)
C8—N5—N6	103.1 (5)	C13—C11—N11	106.1 (4)
C7—N6—C10	130.8 (5)	C13—C11—C19	132.0 (5)
C7—N6—N5	110.7 (5)	N11—C11—C19	121.9 (5)
C10—N6—N5	117.9 (5)	N11—C12—N12	108.3 (5)
C8—N7—H7A	118 (5)	N11—C12—H12A	125.8
C8—N7—H7B	112 (5)	N12—C12—H12A	125.8
H7A—N7—H7B	130 (7)	C11—C13—N12	106.5 (5)
C10—N8—H8A	116 (5)	C11—C13—C14	130.8 (5)
C10—N8—H8B	110 (5)	N12—C13—C14	122.5 (5)
H8A—N8—H8B	134 (7)	C13—C14—S3	113.3 (4)
C3—C1—N1	106.1 (5)	C13—C14—H14A	108.9
C3—C1—C9	131.6 (5)	S3—C14—H14A	108.9
N1—C1—C9	122.2 (5)	C13—C14—H14B	108.9
N1—C2—N2	107.9 (5)	S3—C14—H14B	108.9
N1—C2—H2	126.0	H14A—C14—H14B	107.7
N2—C2—H2	126.0	C16—C15—S3	114.0 (4)
C1—C3—N2	106.0 (5)	C16—C15—H15A	108.8
C1—C3—C4	131.4 (5)	S3—C15—H15A	108.8
N2—C3—C4	122.6 (5)	C16—C15—H15B	108.8
C3—C4—S1	110.1 (4)	S3—C15—H15B	108.8
C3—C4—H4A	109.6	H15A—C15—H15B	107.6
S1—C4—H4A	109.6	N13—C16—C15	111.0 (4)
C3—C4—H4B	109.6	N13—C16—H16A	109.4
S1—C4—H4B	109.6	C15—C16—H16A	109.4
H4A—C4—H4B	108.1	N13—C16—H16B	109.4
C6—C5—S1	114.4 (4)	C15—C16—H16B	109.4
C6—C5—H5A	108.7	H16A—C16—H16B	108.0
S1—C5—H5A	108.7	N13—C17—N14	127.2 (4)
C6—C5—H5B	108.7	N13—C17—N16	126.8 (4)
S1—C5—H5B	108.7	N14—C17—N16	106.0 (4)
H5A—C5—H5B	107.6	N15—C18—N17	126.3 (5)
N3—C6—C5	113.2 (5)	N15—C18—N14	112.5 (4)
N3—C6—H6A	108.9	N17—C18—N14	121.2 (4)
C5—C6—H6A	108.9	C11—C19—H19A	109.5
N3—C6—H6B	108.9	C11—C19—H19B	109.5
C5—C6—H6B	108.9	H19A—C19—H19B	109.5
H6A—C6—H6B	107.8	C11—C19—H19C	109.5
N3—C7—N4	125.6 (5)	H19A—C19—H19C	109.5
N3—C7—N6	128.0 (5)	H19B—C19—H19C	109.5
N4—C7—N6	106.4 (5)	N18—C20—N16	113.0 (4)
N5—C8—N7	125.2 (6)	N18—C20—S4	124.6 (4)
N5—C8—N4	113.3 (5)	N16—C20—S4	122.4 (4)
N7—C8—N4	121.4 (6)	Cl1—Bi1—Cl2	90.41 (4)
C1—C9—H9A	109.5	Cl1—Bi1—Cl3	91.37 (4)
C1—C9—H9B	109.5	Cl2—Bi1—Cl3	95.21 (4)
H9A—C9—H9B	109.5	Cl1—Bi1—Cl4	96.86 (5)
C1—C9—H9C	109.5	Cl2—Bi1—Cl4	89.39 (4)
H9A—C9—H9C	109.5	Cl3—Bi1—Cl4	170.54 (4)
H9B—C9—H9C	109.5	Cl1—Bi1—Cl5	89.31 (5)
N8—C10—N6	113.9 (6)	Cl2—Bi1—Cl5	175.50 (4)
N8—C10—S2	125.2 (5)	Cl3—Bi1—Cl5	89.29 (4)
N6—C10—S2	120.9 (4)	Cl4—Bi1—Cl5	86.18 (5)
C14—S3—C15	103.0 (3)	Cl1—Bi1—Cl6	176.47 (4)
C12—N11—C11	109.9 (5)	Cl2—Bi1—Cl6	88.29 (4)
C12—N11—H11N	116 (5)	Cl3—Bi1—Cl6	85.48 (4)
C11—N11—H11N	134 (5)	Cl4—Bi1—Cl6	86.40 (4)
C12—N12—C13	109.2 (5)	Cl5—Bi1—Cl6	92.24 (4)
C12—N12—H12N	125 (5)	O3—N19—O4	122.6 (5)
C13—N12—H12N	126 (5)	O3—N19—O5	119.0 (4)
C17—N13—C16	125.9 (4)	O4—N19—O5	118.5 (5)
C17—N13—H13N	112 (5)	H11—O1—H12	109 (3)
C16—N13—H13N	122 (5)	H21—O2—H22	105 (3)
			
C8—N5—N6—C7	−0.3 (6)	C18—N15—N16—C17	−1.2 (5)
C8—N5—N6—C10	−172.7 (5)	C18—N15—N16—C20	−176.5 (4)
C2—N1—C1—C3	0.1 (7)	C12—N11—C11—C13	0.2 (6)
C2—N1—C1—C9	177.0 (5)	C12—N11—C11—C19	179.2 (5)
C1—N1—C2—N2	0.3 (7)	C11—N11—C12—N12	−0.3 (6)
C3—N2—C2—N1	−0.5 (7)	C13—N12—C12—N11	0.3 (6)
N1—C1—C3—N2	−0.4 (6)	N11—C11—C13—N12	0.1 (5)
C9—C1—C3—N2	−176.9 (6)	C19—C11—C13—N12	−178.9 (5)
N1—C1—C3—C4	178.3 (5)	N11—C11—C13—C14	174.8 (5)
C9—C1—C3—C4	1.8 (10)	C19—C11—C13—C14	−4.1 (10)
C2—N2—C3—C1	0.6 (6)	C12—N12—C13—C11	−0.2 (6)
C2—N2—C3—C4	−178.2 (5)	C12—N12—C13—C14	−175.6 (5)
C1—C3—C4—S1	−91.1 (6)	C11—C13—C14—S3	−110.4 (6)
N2—C3—C4—S1	87.4 (5)	N12—C13—C14—S3	63.7 (6)
C5—S1—C4—C3	−147.7 (4)	C15—S3—C14—C13	70.6 (5)
C4—S1—C5—C6	−71.8 (5)	C14—S3—C15—C16	−83.8 (5)
C7—N3—C6—C5	−94.9 (6)	C17—N13—C16—C15	−105.3 (6)
S1—C5—C6—N3	−61.7 (6)	S3—C15—C16—N13	−48.5 (6)
C6—N3—C7—N4	−9.9 (9)	C16—N13—C17—N14	0.8 (8)
C6—N3—C7—N6	171.2 (5)	C16—N13—C17—N16	−179.1 (5)
C8—N4—C7—N3	179.7 (6)	C18—N14—C17—N13	178.9 (5)
C8—N4—C7—N6	−1.2 (6)	C18—N14—C17—N16	−1.2 (5)
C10—N6—C7—N3	−8.8 (10)	C20—N16—C17—N13	−4.0 (8)
N5—N6—C7—N3	−180.0 (5)	N15—N16—C17—N13	−178.6 (5)
C10—N6—C7—N4	172.1 (6)	C20—N16—C17—N14	176.1 (5)
N5—N6—C7—N4	0.9 (6)	N15—N16—C17—N14	1.5 (5)
N6—N5—C8—N7	179.9 (6)	N16—N15—C18—N17	−179.7 (6)
N6—N5—C8—N4	−0.5 (7)	N16—N15—C18—N14	0.4 (6)
C7—N4—C8—N5	1.1 (7)	C17—N14—C18—N15	0.5 (6)
C7—N4—C8—N7	−179.3 (6)	C17—N14—C18—N17	−179.3 (5)
C7—N6—C10—N8	−179.0 (6)	C17—N16—C20—N18	−177.0 (5)
N5—N6—C10—N8	−8.3 (8)	N15—N16—C20—N18	−2.8 (6)
C7—N6—C10—S2	2.1 (9)	C17—N16—C20—S4	3.7 (7)
N5—N6—C10—S2	172.8 (4)	N15—N16—C20—S4	177.9 (3)

### Hydrogen-bond geometry (Å, º)

**Table:**

*D*—H···*A*	*D*—H	H···*A*	*D*···*A*	*D*—H···*A*
N1—H1*N*···Cl6^i^	0.89 (4)	2.54 (3)	3.379 (6)	158 (7)
N2—H2*N*···Cl6^ii^	0.88 (5)	2.58 (5)	3.406 (5)	156 (6)
N3—H3*N*···S2	0.89 (5)	2.23 (6)	3.019 (6)	148 (7)
N4—H4*N*···O2	0.91 (7)	1.74 (7)	2.608 (7)	160 (7)
N7—H7*A*···S2^iii^	0.91 (6)	2.46 (7)	3.296 (6)	155 (6)
N7—H7*B*···O1^iv^	0.90 (4)	1.97 (5)	2.842 (8)	162 (6)
N8—H8*A*···Cl5^iv^	0.89 (5)	2.83 (6)	3.550 (5)	140 (5)
O1—H11···Cl5	0.84 (6)	2.39 (6)	3.138 (5)	149 (6)
N11—H11*N*···O1^v^	0.89 (2)	1.83 (3)	2.703 (6)	170 (9)
O1—H12···Cl4^vi^	0.85 (6)	2.27 (5)	3.116 (5)	172 (7)
N12—H12*N*···O3^ii^	0.88 (6)	1.99 (7)	2.835 (6)	162 (6)
N12—H12*N*···O4^ii^	0.88 (6)	2.58 (6)	3.307 (8)	142 (5)
N13—H13*N*···S4	0.88 (3)	2.27 (6)	3.025 (4)	144 (7)
N14—H14*N*···O3^vii^	0.87 (6)	1.91 (6)	2.772 (5)	175 (5)
N14—H14*N*···O5^vii^	0.87 (6)	2.43 (8)	2.960 (7)	120 (7)
N17—H17*A*···S4^iii^	0.87 (6)	2.60 (6)	3.423 (5)	159 (6)
N17—H17*B*···Cl3^iii^	0.87 (3)	2.52 (5)	3.337 (5)	158 (6)
N18—H18*A*···Cl2	0.87 (5)	2.56 (7)	3.304 (5)	144 (5)
N18—H18*B*···Cl3	0.88 (4)	2.59 (7)	3.332 (5)	143 (7)
O2—H21···Cl4	0.86 (4)	2.62 (6)	3.326 (5)	139 (6)
O2—H21···S2^iii^	0.86 (4)	2.59 (8)	3.168 (5)	126 (7)
O2—H22···O4	0.86 (3)	2.52 (5)	3.227 (7)	139 (7)
O2—H22···O5	0.86 (3)	1.88 (5)	2.717 (6)	162 (9)

Symmetry codes: (i) −*x*+2, −*y*+1, −*z*+1; (ii) −*x*+1, −*y*+1, −*z*+1; (iii) *x*−1, *y*, *z*; (iv) −*x*+1, −*y*+1, −*z*; (v) −*x*+1, −*y*, −*z*+1; (vi) *x*+1, *y*, *z*; (vii) −*x*, −*y*+1, −*z*+1.
